# Residential aircraft and road noise exposure, cognition, and Alzheimer’s dementia in a US cohort of older adults

**DOI:** 10.1097/EE9.0000000000000518

**Published:** 2026-08-10

**Authors:** Stephanie T. Grady, Ryan M. Andrews, Junenette L. Peters, Sara D. Adar, Jonathan I. Levy, Todd Beck, Klodian Dhana, Pankaja Desai, Bernd J. Haupt, Joel D. Kaufman, Adam A. Szpiro, Robert S. Wilson, Kumar B. Rajan, Jennifer Weuve

**Affiliations:** a Department of Environmental Health, Boston University School of Public Health, Boston, Massachusetts; b Department of Epidemiology, Boston University School of Public Health, Boston, Massachusetts; c Department of Epidemiology, University of Michigan School of Public Health, Ann Arbor, Michigan; d Department of Internal Medicine, Rush Institute for Healthy Aging, Rush University Medical Center, Chicago, Illinois; e Earth and Environmental Systems Institute, Pennsylvania State University, University Park, Pennsylvania; f Departments of Environmental and Occupational Health Sciences, Epidemiology, and Medicine, University of Washington, Seattle, Washington; g Department of Biostatistics, University of Washington School of Public Health, Seattle, Washington; h Department of Neurological Sciences and Department of Psychiatry and Behavioral Snociences, Rush Alzheimer’s Disease Center, Rush University Medical Center, Chicago, Ilinois

## Abstract

**Background:**

There is limited evidence of the associations between transportation noise and dementia risk. We examined associations of road and aircraft noise with cognitive function, cognitive decline, and Alzheimer’s dementia (AD) in a cohort of older adults.

**Methods:**

We set our study in the Chicago Health and Aging Project, a longitudinal cohort of community-dwelling older adults from four Chicago, Illinois, neighborhoods, followed from 1993 to 2012. Every 3 years, participants underwent in-home cognitive assessments (episodic memory, perceptual speed, global cognition). A stratified random sample underwent clinical evaluation for AD diagnosis. We estimated 5-year time-weighted day–night average sound levels before baseline for road and, in analyses, we considered exploratory because of a limited exposure range, aircraft noise. We fit covariate-adjusted linear mixed models to estimate associations with cognitive performance and decline, and a multiple logistic regression model weighted by the sampling design to estimate incident AD odds ratios.

**Results:**

Of the 10,681 participants, an interquartile range increment in road noise (8.5 dBA) corresponded to a 0.06-SD unit lower baseline global cognitive score (95% confidence interval: −0.08, −0.03); there were no differences in cognitive decline. In exploratory analyses limited by power, higher aircraft noise was associated with a higher baseline score yet a faster rate of cognitive decline. Among 2147 participants evaluated for dementia (460 cases), road noise was not noticeably associated with greater AD risk, as there was substantial uncertainty (odds ratio per interquartile range = 1.17 [95% confidence interval: 0.86, 1.59]).

**Conclusion:**

We observed mixed associations of transportation noise with cognitive functioning and dementia across noise sources. These findings underscore the need to better characterize noise sources relevant to the cognitive health of older adults.

**What this study adds**

We examined associations of transportation noise with cognitive performance, Alzheimer’s dementia (AD), and cognitive decline among >10,000 participants from the Chicago Health and Aging Project, a cohort of older adults (1993–2012). Higher road noise levels were associated with lower baseline cognitive performance yet were not associated with cognitive decline. Conversely, higher aircraft noise levels were associated with higher baseline performance yet faster rates of cognitive decline. We did not observe associations with AD. Overall, these mixed findings suggest that associations between noise and cognitive outcomes may vary by source, underscoring the need for improved characterization of environmental noise in US cities.

## Introduction

Over 104 million Americans (approximately one in three individuals) are exposed to high levels of community noise (residential, nonoccupational exposures).^1^ There is growing evidence linking noise to poor cognition^2–7^ and dementia^2–4,8–10^ in older adults globally, as noise could plausibly increase risk by eliciting stress responses, including elevating blood pressure and inducing disturbances in sleep.^11,12^ These biological changes could affect cognitive health by increasing oxidative damage and inflammation, dysregulating neurohormone secretion and brain waste clearance, and leading to subsequent neurodegeneration.^12–14^ Among epidemiological studies on noise, few have examined associations with the rate of cognitive decline,^4,6^ as the manifestation of prodromal and clinical dementia. Moreover, few studies have been conducted on source-specific noise in the United States.^3,4^

The effects of community noise from specific sources (e.g., road, aircraft) are of interest as each source has distinct patterns in frequency, intermittency, and intensity—patterns that may vary in their pertinence to dementia etiology. Identifying these source-specific associations with dementia may inform noise-exposure reduction strategies that better target health benefits. Although there is an extensive body of research into the effects of noise on annoyance, sleep, and cardiovascular endpoints,^15–17^ there have been fewer evaluations of the effects on cognitive health in older adults. Furthermore, the number of people in the United States living with dementia, a condition now presumed to have myriad non-genetic determinants,^18^ is set to reach 12.7 million by 2050,^19^ in the absence of population-wide approaches to prevention and treatment.

In the Chicago Health and Aging Project (CHAP), we previously conducted a study investigating the relation between community noise and baseline cognitive performance, rate of cognitive decline, and the prevalence of Alzheimer’s disease (AD) dementia.^4^ Among 5227 participants, each 10-A-weighted decibel (dBA) increment in community noise corresponded to a 0.04-SD unit lower (95% confidence interval [CI]: −0.11, 0.04) global cognitive score at baseline, along with a 29% higher odds (95% CI: 1.08, 1.55) of prevalent AD over all observation waves.^4^ No associations were found with cognitive decline. Since study completion, we have developed period-specific measures of exposure to transportation-specific noise for cohort participants. Understanding the specific contributions of road and aircraft noise is vital, as regulating and/or mitigating noise at specific source points represents a more realistic solutions-based approach to addressing noise concerns. Therefore, this study aimed to estimate the associations of period-specific road and aircraft noise exposure with cognitive performance, cognitive decline, and incident dementia in older adults in CHAP. Additionally, we conducted secondary analyses to estimate associations between community noise and our cognitive outcomes and to compare with source-specific analyses.

## Methods

### Study sample

We conducted our study in CHAP, a longitudinal community cohort of older adults in Chicago, Illinois, from 1993 to 2012. More information on study recruitment can be found in eText 1, https://links.lww.com/EE/A455. In total, CHAP enrolled 10,802 participants residing across a ~15-square-mile (38.8 km^2^) area, with staggered cohort entry and varying follow-up durations.^4^ At baseline and approximately every 3 years, research staff conducted in-home participant interviews, asking structured questions about health and health behaviors. Participants also underwent cognitive assessments at each interview. The final analytic sample included participants who had at least one pair of noise and outcome data and were not missing any covariate information.

### Noise-exposure assessment

Primary exposures of interest were road and aircraft noise over the long term; however, we also sought to re-examine community noise^4^ as we had broadened our study time frame from 1999–2012 to 1993–2012 (given availability of source-specific noise exposures). We measured these long-term exposures using 5-year time-weighted averages of each noise exposure anchored to baseline visits (cognition) or clinical evaluations (incident dementia), using the *intervalaverage* package in R version 4.3.2 (Vienna, Austria). The choice of a 5-year exposure window represents a balance between available data and the goal of representing long-term exposure. Participant residential addresses were geocoded in ESRI ArcGIS 9.3 using the Tele Atlas Dynamap 2000 v. 16.1 road network (Boston, MA).

#### Aircraft noise

Aircraft day–night average sound level (DNL) contour maps were obtained from the John A. Volpe National Transportation Systems Center in 5-year increments from 1995 to 2015.^20,21^ DNL represents an A-weighted equivalent sound level (*L*_eq_) over 24 hours, with a 10-dBA penalty for nighttime hours (10:00 pm–7:00 am). Contours were modeled using the Aviation Environmental Design Tool to estimate DNL in 1-dBA increments down to 45 dBA and at a 0.1 nautical mile (~185.2 m) spatial resolution.^20–22^ For areas with aircraft noise <45 dBA, we assigned values of 40 dBA, as this was below the minimum noise level;^23^ these estimates were used to create 5-year time-weighted averages, similar to previous work in this cohort.^4,24^ For years in which estimates were unavailable, we interpolated time-weighted estimates using the closest previous and following years of data. For periods before 1995, we assigned estimates based on those from 1995. Given the limited range of exposure and small number of highly exposed participants according to this measure, we considered aircraft noise analyses as exploratory.

#### Road noise

Annualized road DNL for Chicago was estimated using CadnaA noise propagation software, with annual average daily traffic (AADT) data from the Illinois Department of Transportation (DOT) (http://apps.dot.illinois.gov/gist2/) accessed in 2018 and the Federal Highway Administration Transportation Traffic Noise Model calculation method for modeling. Transportation Traffic Noise Model uses AADT and other inputs (e.g., vehicle type and speed) to estimate noise on a 100 m × 100 m grid. Estimates were modeled for 1996 (due to the availability of AADT data) and every 5 years from 2000 to 2015. Like aircraft noise, for years in which data were unavailable, we interpolated time-weighted estimates based on the closest previous and following years of data. For periods before 1996, we assigned estimates based on those from 1996.

#### Community noise

We used community noise estimates examined in prior CHAP studies,^4,24^ which represented the totality of outdoor daytime noise. Briefly, this metric was based on spatial predictions from a universal kriging model built using noise measurements from a 2006–2007 Chicago sampling campaign across 136 locations during nonrush-hour periods; as such, this metric does not capture nighttime noise.^25^ This prediction model included land use and other geographic covariates, such as proximity to major roadways and bus stops.^25^ In 2016, the team re-measured noise levels, which were similar to those from a decade earlier.^4^ Therefore, we assumed levels at a given location were consistent throughout the study period in the Chicago area.

### Outcome assessment

#### Cognitive function

Our cognitive measures of interest were perceptual speed, episodic memory, and overall global cognitive performance. At each 3-year interview, CHAP participants underwent brief cognitive assessments comprised of four tests: the oral version of the Symbol Digit Modalities Test,^26,27^ immediate and delayed recall assessments within the East Boston Memory Test,^28^ and the Mini-Mental State Examination (MMSE).^27,29^ To facilitate comparisons across cognitive performance measures, we converted all raw test scores to z-scores, using the baseline means and SDs (eTable 1 https://links.lww.com/EE/A455). For perceptual speed, we used the *z*-score calculated from the Symbol Digit Modalities Test. For episodic memory, we averaged *z*-scores from the immediate and delayed recall sections of the East Boston Memory Test and then transformed this average to the standard normal distribution.^4^ For global cognitive performance, we averaged *z*-scores from the Symbol Digit Modalities Test, East Boston Memory Test, and MMSE; this average was further transformed to the standard normal distribution.^4^

#### Incidence of Alzheimer’s dementia

At each follow-up wave, ~10% of the sample was selected for further clinical evaluation through stratified random sampling, resulting in one-third of the CHAP cohort receiving ≥1 clinical evaluation.^4,30^ This process randomly selected different sets of participants at each study wave within strata of age, sex, race, and change in cognitive function from the previous assessment to efficiently sample cases of dementia to increase statistical power for risk factor analyses without bias.^4,30^ If a participant was selected and underwent a clinical evaluation more than once, they were retained in our study until they received a dementia diagnosis. These participants underwent further neuropsychological testing administered by trained examiners blinded to sampling categories.^30^ A board-certified neuropsychologist made final determinations on cognitive impairment based on the results of five domains: attention, language, memory, orientation, and perception.^30^ Additionally, trained clinical staff obtained a detailed medical history and conducted structured neurological examinations compatible with NIH Stroke Scale^31^ and Stroke Data Bank^32^ guidelines. A clinical diagnosis of dementia was determined by the study neurologist and defined as the loss of cognitive function and impairment in ≥2 cognitive domains.^30^ A dementia diagnosis was further classified as probable AD, using National Institute of Neurological and Communicative Disorders and Stroke and the Alzheimer’s Disease and Related Disorders Association criteria.^33^ Participants who met these criteria and had a co-existing condition impairing cognition also met the definition of AD.^30^ About 93% of dementia cases met the criteria for AD alone or mixed with another dementia.^4^

### Standard protocol approvals and consent

CHAP and this study were approved by the Institutional Review Boards at Rush University Medical Center, Boston University, and the University of Michigan. All participants provided written informed consent.

### Covariates

We adjusted our analyses for hypothesized sources of confounding or covariates thought to influence precision, specifically: time in study, age, race, educational attainment, apolipoprotein E (*APOE*) ε4 allele carriership, nitrogen oxide (NO_*x*_) concentration, and neighborhood-level socioeconomic status (nSES). Time in study was calculated as the number of years between the current and first visits. Age was calculated by subtracting participant birthdates from visit dates in years. Race was categorized as a binary variable (Black, not Black) from baseline self-reported responses to a question identical to that on the 1990 U.S. Census and based on spatial patterning of populations within the region^30^ and rates of dementia between populations.^34^ Sex was also captured as a binary variable (female, male) through baseline self-report, along with educational attainment. The *APOE* ε4 genotype was determined using the hME Sequenom MassARRAY platform and was included as a precision variable in our analyses. We used outdoor NO_*x*_ as a surrogate of localized traffic-related air pollution over the same period as noise, creating 5-year time-weighted averages anchored to participants’ visit dates. NO_*x*_ concentrations were predicted at each residence every 2 weeks from 1999 using a spatiotemporal model developed for Chicago.^35,36^ For years before 1999, we carried backward 1999 estimates for each location. For nSES, we created a composite score based on the average of *z*-scores for several U.S. Census variables within the CHAP area at the block group level in 2000.^37^ Higher nSES scores indicated greater affluence. Although nSES estimates were specific to 2000, we created 5-year time-weighted estimates for individuals who moved residences between visits. We did not adjust for other factors that may have been influenced by noise exposure or cognition (e.g., blood pressure, health status/quality of life).

### Statistical methods

In this study, we estimated associations of each noise measure with baseline cognitive performance, cumulative incidence of AD and all-cause dementia, and cognitive change over time. Aircraft DNL was analyzed categorically (<45, 45–49, 50–54, ≥55 dBA), given the large number of individuals with exposure no more specific than <45 dBA. Road and community noise were analyzed continuously, as we previously did not find evidence of nonlinear associations when visually examining penalized splines for the association of road and community noise with cognition.

To estimate associations between noise and cognitive performance and decline, we fitted multivariable-adjusted linear mixed-effects models, using years since baseline as the time scale. The association of noise with baseline cognitive function corresponded to the coefficient for noise; the association of noise with the rate of cognitive change corresponded to the cross-product between noise and time (eText 1, https://links.lww.com/EE/A455). We adjusted these analyses for age and calendar year at baseline, sex (female, male), race (Black, not Black), education (<high school, high school, >high school), averaged NO_*x*_ concentration (continuous), nSES (continuous), and cross-products of each covariate with time since baseline. We included a random intercept to account for differences in baseline cognitive scores between participants. We report mean differences in *z*-scores, reflecting SDs in cognitive performance, per interquartile range (IQR) increment in noise exposure over 5 years.

To estimate associations with incident dementia among participants free at baseline, we fit logistic regression models accounting for the random stratified sample design,^30^ adjusting for calendar year and age at clinical evaluation, race, sex, *APOE* ε4 allele carriership, education, NO_*x*_, and nSES. As AD and all-cause dementia were diagnosed from random stratified subsamples, we weighted associations to the distribution of the overall CHAP cohort. For reporting, we transformed coefficients into 3-year cumulative incidence odds ratios per IQR increment in noise exposure. We evaluated the precision of all estimates using widths of 95% CIs.

In secondary analyses, we fit models to investigate associations between community noise and our outcomes, similar to analyses with source-specific noise, to replicate our previous work^4^ over a wider time period. Lastly, we evaluated the potential for selective attrition over follow-up (eFigure 1 and eText 2, https://links.lww.com/EE/A455).

## Results

### Descriptive statistics

The study consisted of 10,681 participants who contributed a range of one to six visits, with a mean (SD) follow-up period of 5.6 (5.0) years. Participants were, on average, 73.3 years old at baseline, with 62% female and 63% identifying as Black (Table 1). Our analysis examined incident AD and all-cause dementia in 2149 participants.

**Table 1.** Distribution of 5-year time-weighted noise levels at baseline by CHAP participant characteristics^a^

	All participants N (%)	Aircraft DNL, in dBA N (%)				Road DNL, in dBA N (%)^b^			
		<45	45–49	50–54	≥55	<53	53–56	57-60	≥61
Number of participants	10,681 (100)	4,464 (41.8)	2,988 (27.9)	2,486 (23.3)	743 (7.0)	2,843 (26.6)	2,855 (26.7)	2,329 (21.8)	2,654 (24.9)
Age, years									
<75	7,257 (67.9)	2,722 (61.0)	2,027 (67.8)	1,903 (76.6)	605 (81.4)	1,890 (66.5)	1,918 (67.2)	1,614 (69.3)	1,835 (69.1)
75–84	2,492 (23.3)	1,237 (27.7)	698 (23.4)	455 (18.3)	102 (13.7)	713 (25.1)	656 (23.0)	516 (22.2)	607 (22.9)
≥85	932 (8.7)	505 (11.3)	263 (8.8)	128 (5.2)	36 (4.9)	240 (8.4)	281 (9.8)	199 (8.5)	212 (8.0)
Sex									
Female	6,571 (61.5)	2,821 (63.2)	1,822 (61.0)	1,474 (59.3)	454 (61.1)	1,724 (60.6)	1,747 (61.2)	1,424 (61.1)	1,676 (63.2)
Male	4,110 (38.5)	1,643 (36.8)	1,166 (39.0)	1,012 (40.7)	289 (38.9)	1,119 (39.4)	1,108 (38.8)	905 (38.9)	978 (36.9)
Race									
Black	6,733 (63.0)	2,143 (48.0)	1,992 (66.7)	2,061 (82.9)	537 (72.3)	1,214 (42.7)	1,835 (64.3)	1,674 (71.9)	2,010 (75.5)
Not Black^c^	3,948 (37.0)	2,321 (52.0)	996 (33.3)	425 (17.1)	206 (27.7)	1,629 (57.3)	1,020 (35.7)	655 (28.1)	644 (24.3)
*APOE* ε4 carrier									
Yes	1,920 (18.0)	742 (16.6)	544 (18.2)	438 (17.6)	196 (26.4)	467 (4.4)	554 (19.4)	414 (17.8)	485 (18.3)
No	3,876 (36.3)	1,632 (36.6)	1,037 (34.7)	891 (35.8)	316 (42.5)	1,119 (39.4)	1,037 (36.3)	834 (35.8)	886 (33.4)
Missing	4,885 (45.7)	2,090 (46.8)	1,407 (47.1)	1,157 (46.5)	231 (31.1)	1,257 (44.2)	1,264 (44.3)	1,081 (46.4)	1,283 (48.3)
Education, years									
<12	3,346 (31.3)	1,286 (28.8)	943 (31.6)	950 (38.2)	167 (22.5)	672 (23.6)	881 (30.9)	769 (33.0)	1,024 (38.6)
12	3,139 (29.4)	1,318 (29.5)	975 (30.9)	704 (28.3)	193 (26.0)	838 (29.5)	848 (29.7)	706 (30.3)	747 (28.2)
≥12	4,196 (39.3)	1,785 (41.7)	1,196 (37.5)	832 (33.5)	383 (51.6)	1,333 (46.9)	1,126 (39.4)	854 (36.7)	883 (33.3)
Community *L*_eq_, dbA									
<55.4	5,326 (49.9)	2,003 (44.9)	1,498 (50.1)	1,442 (58.0)	383 (51.2)	1,709 (63.5)	1,805 (59.9)	1,206 (51.8)	606 (22.8)
≥55.4	5,326 (49.9)	2,439 (54.6)	1,485 (49.7)	1,042 (41.9)	360 (48.5)	1,023 (36.0)	1,141 (40.0)	1,122 (48.2)	1,020 (76.9)
Missing	29 (0.3)	22 (0.5)	5 (0.2)	2 (0.1)	0 (0)	15 (0.53)	5 (0.18)	1 (0.04)	8 (0.30)
NO_*x*_, ppb^d^									
<45.6	5,341 (50.0)	2,616 (58.6)	1,308 (43.8)	879 (35.4)	538 (72.4)	2,114 (74.4)	1,519 (53.2)	920 (39.5)	788 (29.7)
≥45.6	5,340 (50.0)	1,848 (41.4)	1,680 (56.2)	1,607 (64.6)	205 (27.6)	729 (25.6)	1,336 (46.8)	1,409 (60.5)	1,866 (70.3)
nSES, average *z*-score^d^									
<0.27	5,320 (49.8)	964 (21.6)	1,885 (63.1)	1,984 (79.8)	487 (65.6)	924 (32.5)	1,493 (52.3)	1,305 (56.0)	1,598 (60.2)
≥0.27	5,361 (51.2)	3,500 (78.4)	1,103 (36.9)	502 (20.2)	256 (34.5)	1,919 (67.5)	1,362 (47.7)	1,024 (44.0)	1,056 (39.8)

a Column percentages: Each of these values represents the percentage of participants exposed to a particular noise group who have the characteristic category of interest. Percentages may not add up to 100% due to rounding.

b Cut-points represent quartiles of road noise.

c There were relatively few participants that identified as neither Black nor White: 13 participants identified as American Indian, 1 as Aleut, and 19 as Asian or Pacific Islander. In total, these participants contributed to <1% of the sample. Although not ideal, we decided to dichotomize race based on identifying as either Black or non-Black.

d Cut-point represents the median of the participant characteristic.

*APOE* ε4 indicates apolipoprotein E ε4 allele; dBA, A-weighted decibels; CHAP, Chicago Health and Aging Project; DNL, day–night average sound level; *L*_eq_, equivalent continuous sound pressure level; N, sample size; NO_x_, nitrogen oxide concentration; nSES, neighborhood socioeconomic status; ppb, parts per billion.

**Figure 1.**
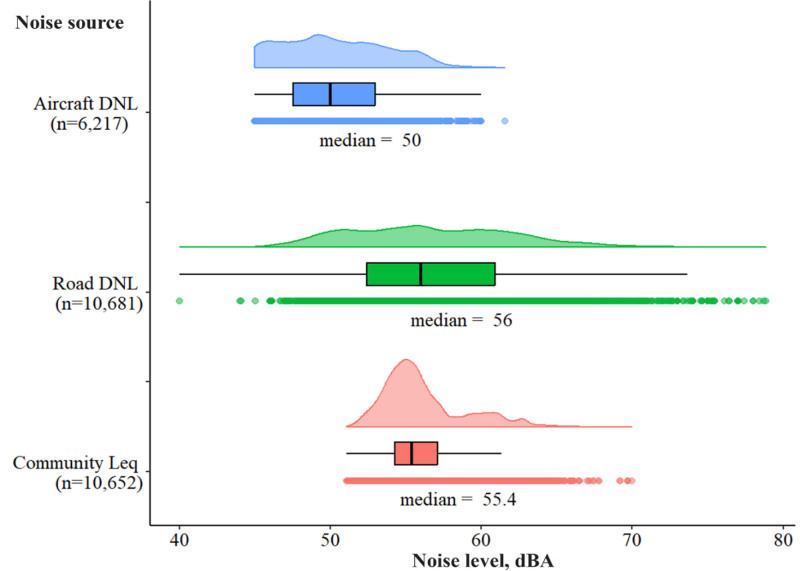
Distribution of 5-year time-weighted averages of noise exposure prior to baseline by source in the CHAP cognitive assessment sample, illustrated using rain cloud plots. Raincloud plots consist of three descriptive plots (in order of presentation: half-violin plot, boxplot, dot density plot) that characterize the distribution of values for each noise measure. The half-violin plot describes the distribution of values within the sample using kernel density smoothing and provides context for the shape and spread of the data. The boxplot characterizes the data with the vertical line in the middle of the box representing the median value, the lateral ends of the box representing the first (left) and third (right) quartiles, and the left and right ends of the whiskers representing 1.5 times the lower or upper quartiles, respectively. The dot density plot represents the number of raw data points vertically drawn for each value in the sample. Aircraft DNL distribution is based on values ≥45 dBA, which comprises 58.2% of participants; the remaining 41.8% of participants were exposed to aircraft DNL <45 dBA. CHAP indicates Chicago Health and Aging Project; dBA, A-weighted decibels; DNL, day–night average sound level; *L*_eq_, equivalent continuous sound pressure level; n, sample size.

**Figure 2.**
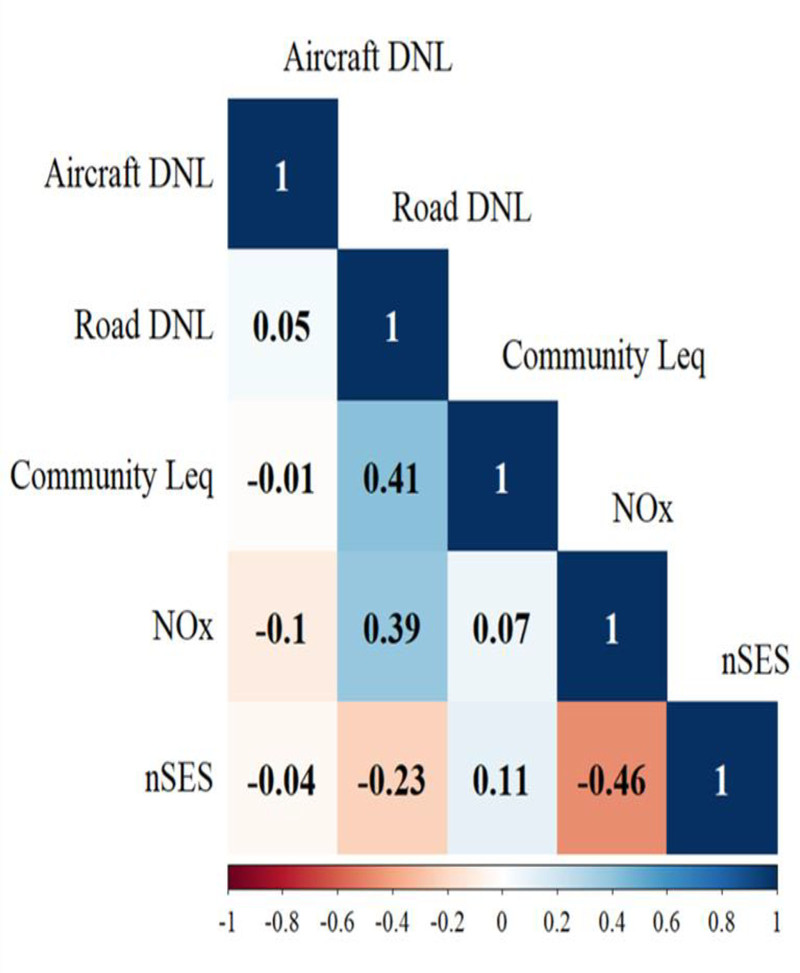
Spearman correlations between environmental exposures and neighborhood socioeconomic status among 10,681 participants at study baseline. DNL indicates day–night average sound level; *L*_eq_, equivalent continuous sound pressure level; NO_*x*_, nitrogen oxide concentration; nSES, neighborhood socioeconomic status.

Baseline noise varied by source, with median (IQR) levels of 50.0 (5.5) dBA for aircraft, 56.0 (8.5) dBA for road, and 55.4 (2.6) dBA for community noise (Figure 1). Approximately 41.8% of participants were exposed to aircraft noise <45 dBA, 28.0% to 45–49 dBA, 23.3% to 50–54 dBA, and 7.0% to ≥55 dBA, with a maximum level of 61.6 dBA. Road noise had the widest range, from 40 to 78.8 dBA. As expected for this geographic area, road noise was the dominant transportation noise source. Regarding relations between noise measures, aircraft was not correlated with road or community noise, yet road and community noise were moderately and positively correlated with each other (Figure 2). Source-specific noise was associated with other participant characteristics: individuals exposed to higher aircraft and road noise were more likely to identify as Black and reside in areas of lower nSES (Figure 2 and Table 1). Among those exposed to the highest aircraft noise category, we observed higher proportions of younger participants (<75 years); yet among those exposed to the highest road noise category, there were no discernible differences in age (Table 1).

### Cognitive performance at baseline

**Figure 3.**
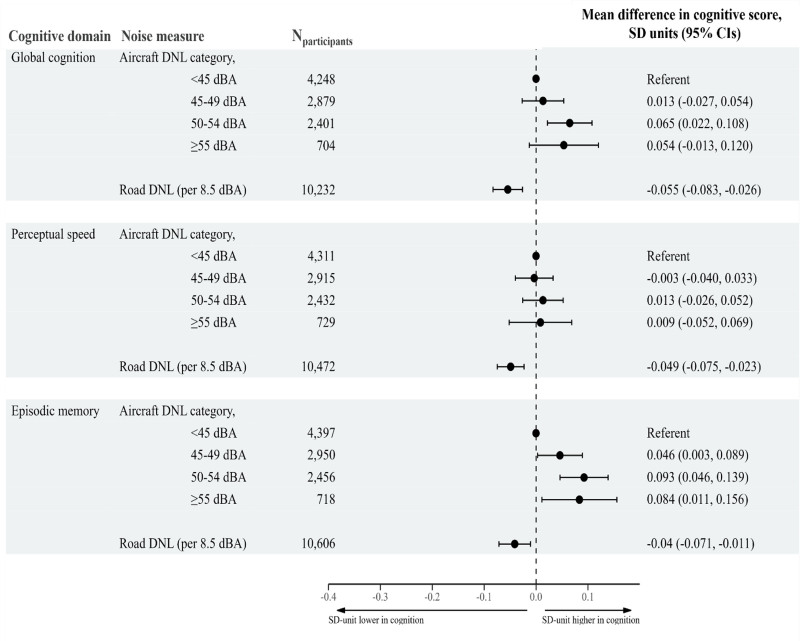
Multivariable-adjusted mean differences in cognitive performance at baseline with increasing levels of aircraft and road noise. Adjusted for baseline year of visit, baseline age, race, sex, education level, averaged NO_*x*_ concentration, nSES score, time since baseline, and cross-products of each covariate and time since baseline. Perceptual speed and episodic memory domains represent subscores of global cognitive performance. CI indicates confidence interval; dBA, A-weighted decibels; DNL, day–night average sound level; N_participants_, number of participants; NO_*x*_, nitrogen oxide; nSES, neighborhood socioeconomic status; SD, standard deviation.

Multivariable-adjusted associations between noise and baseline cognitive performance differed by source (Figure 3). Aircraft noise findings demonstrated positive, nonlinear associations with global cognition, with higher noise associated with better cognition (following patterns observed for episodic memory). There were no associations with perceptual speed. By contrast, higher road noise was inversely associated with all cognitive domains. Associations of noise with other subscore measures (i.e., immediate recall, delayed recall, MMSE) yielded similar patterns (eTable 2, https://links.lww.com/EE/A455).

### Rate of change in cognitive performance

**Figure 4.**
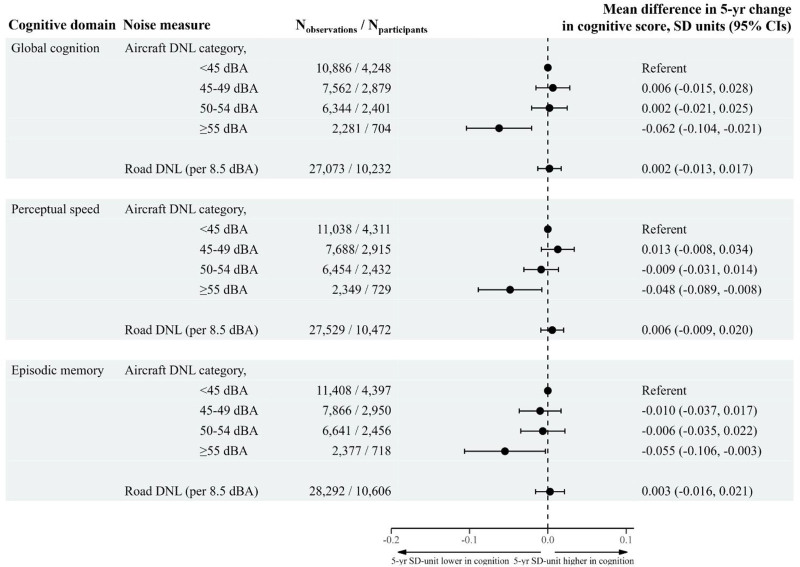
Multivariable-adjusted mean differences in the rate of change in cognitive performance with increasing levels of aircraft and road noise. Adjusted for baseline year of visit, baseline age, race, sex, education level, averaged NO_*x*_ concentration, nSES score, time since baseline, and cross-products of each covariate and time since baseline. Perceptual speed and episodic memory domains represent subscores of global cognitive performance. CI indicates confidence interval; dBA, A-weighted decibels; DNL, day–night average sound level; N_obs_, number of observations; N_participants_, number of participants; NO_*x*_, nitrogen oxide; nSES, neighborhood socioeconomic status; SD, standard deviation.

Participants exposed to the highest (compared with the lowest) aircraft noise category experienced faster declines in all cognitive measures (Figure 4). For example, those exposed to aircraft noise ≥55 dBA experienced, on average, a 0.062-SD faster decline in global cognition every 5 years past baseline (95% CI: −0.104, −0.021) than those exposed to <45 dBA. However, we did not observe associations in lesser exposed groups (i.e., 45–49 dBA, 50–54 dBA). Road noise was not associated with the rate of change in any of the cognitive outcomes. Analogous associations with the rate of change in other cognitive subscores were not meaningfully different from associations with global cognition, episodic memory, and perceptual speed (eTable 3, https://links.lww.com/EE/A455).

### Alzheimer’s and all-cause dementia

Among individuals who underwent further clinical evaluation, 19.9% (n = 428) and 21.4% (n = 460) received AD and all-cause dementia diagnoses, respectively (Table 2). The weighted, multivariable-adjusted associations between aircraft noise and AD were nonmonotonic yet imprecise. For example, compared to the lowest aircraft noise category, the AD cumulative incidence OR was 1.61 (95% CI: 0.93, 2.81) for those exposed to 50–54 dBA, but only 1.03 (95% CI: 0.56, 1.89) for those exposed to >55 dBA. For every IQR increment in road noise (8.5 dBA), we observed a 17% higher cumulative odds of incident AD (95% CI: 0.86, 1.59). Associations with all-cause dementia were similar to those for AD.

**Table 2.** Weighted^a^ multivariable-adjusted^b^ cumulative odds ratios of incident Alzheimer’s and all-cause dementia with increasing level of aircraft, road, and community noise

Noise measure (per IQR)	Alzheimer’s dementia		All-cause dementia	
	Cases/non-cases	Odds Ratio (95% CI)	Cases/non-cases	Odds ratio (95% CI)
Aircraft DNL category, dBA				
<45	178/ 733	Referent	190/ 721	Referent
45–49	98/ 470	0.96 (0.60, 1.53)	109/ 459	0.98 (0.62, 1.55)
50–54	102/ 346	1.61 (0.93, 2.81)	108/ 340	1.62 (0.94, 2.80)
≥55	50/ 172	1.03 (0.56, 1.89)	53/ 169	1.05 (0.58, 1.90)
Road DNL (per IQR, 8.5 dBA)	428/ 1,721	1.17 (0.86, 1.59)	460/ 1,689	1.17 (0.87, 1.58)
Community *L*_eq_ (per IQR, 2.6 dBA)	422/ 1,680	1.02 (0.85, 1.21)	453/ 1,649	1.02 (0.86, 1.21)

a Weighted to the distribution of the entire CHAP cohort.

b Adjusted for year of clinical evaluation, age, *APOE ε4* carriership, race, sex, education level, averaged NO_x_ concentration, and nSES score.

*APOE* ε4 indicates apolipoprotein E ε4 allele; CI, confidence interval; dBA, A-weighted decibels; DNL, day–night average sound level; IQR, interquartile range; *L*_eq_, equivalent continuous sound pressure level; NO_x_, nitrogen oxide; nSES, neighborhood socioeconomic status.

### Community noise

In secondary analyses, we investigated associations between community noise and baseline cognitive performance and its rate of change over time (eTable 4, https://links.lww.com/EE/A455). For each IQR (2.6 dBA) increment in community noise, we observed a 0.070-SD lower (95% CI: −0.088, −0.054) baseline global cognitive score. When expressed with the same dBA contrast as road noise (8.5 dBA), the association with community noise was comparably stronger. Community noise was also inversely associated with individual cognitive domains at baseline; yet, overall, no distinct associations were observed with cognitive decline across any domain, similar to prior results.^4^ Community noise was also not strongly associated with incident AD or all-cause dementia.

### Attrition bias

Among participants in this study, approximately 23.2% discontinued participation (due to death or loss to follow-up) before study completion in 2012. The odds of attrition were 5% higher among those exposed to aircraft noise 45–49 dBA compared with those <45 dBA; for those exposed to aircraft noise 50–54 dBA and ≥55 dBA, there were 1% and 4% lower odds of attrition, respectively (eTable 5, https://links.lww.com/EE/A455). We observed a 1% higher odds of attrition for every 1-dBA increase in road noise. Joint associations of noise exposure and cognitive performance with attrition were similarly weak (eTable 6, https://links.lww.com/EE/A455).

## Discussion

In this study, we observed varying patterns in associations of environmental noise exposure with cognitive performance, incident dementia, and cognitive decline. Analyses of road noise and cognitive outcomes yielded results in the hypothesized direction: those exposed to higher levels of road noise had lower baseline cognitive scores, with differences roughly equivalent to differences between participants who were approximately 1 year apart in age. Exposures to road noise also broadly corresponded to a higher incidence of AD; however, these estimates did not translate to associations with cognitive decline over time. In contrast, exploratory associations with aircraft noise were not consistent across cognitive outcomes, both in direction and magnitude.

This study extends previous research on community noise and cognition in this cohort^4^ in important ways. First, we introduced source-specific transportation measures (road and, to some extent, aircraft DNL) to better characterize differences of each noise source with dementia-related outcomes, accounting for nighttime exposures. Because transportation noise data were available starting in 1995/1996, we were able to leverage an additional 5 years of CHAP outcome data for the present study. This longer time frame also enabled us to progress from examining predicted AD prevalence (as reported in the previous study^4^) to examining incident AD.

Several patterns in our findings merit note. Road noise was the dominant source of transportation noise exposure in this cohort (e.g., 61% of participants were exposed to road noise ≥55 dBA). This finding was not surprising: community and road noise were moderately correlated and consistent with reports identifying road noise as the most prevalent residential/community noise exposure.^38,39^ Additionally, community *L*_eq_ from land-use regression models was estimated using short-term 5-minute sound measurements; therefore, we were less able to capture intermittent sounds, such as those from aircraft or construction. Lastly, we observed little evidence that road noise and cognitive performance jointly influenced attrition, similar to our previous analyses of air pollution (i.e., NO_*x*_) and cognition.^40^ Although neither road noise nor NO_*x*_ was associated with cognitive decline in our studies, we did find adverse associations of road noise with baseline cognition. Yet when previous NO_*x*_ analyses were evaluated for pre-enrollment selection bias, results indicated potential differential survivorship.^40^

In terms of aircraft noise, we were limited in our ability to robustly quantify its effects across a full range of exposure experienced in the United States, as only 7% were exposed to ≥55 dBA, demonstrating distinct spatiotemporal patterns of exposure (i.e., flight paths). Additionally, aircraft noise exposure could not be estimated at <45 dBA; therefore, we imputed these levels as 40 dBA. Our findings were generally insensitive to this choice, with similar results using an imputation of 44 dBA; however, more spatially resolved estimates at lower levels could have reduced exposure error. Furthermore, differential participant selection into the cohort may be relevant: contrary to expectation, progressively higher aircraft noise was associated with better performance at baseline (primarily episodic memory). Yet, exposure to the highest noise category was associated with faster decline. These mixed results may have been influenced by differential participation favoring those least susceptible to the adverse effects of noise, biasing estimates on baseline cognition upwards (and downwards on dementia incidence).^40–42^ For example, if individuals with higher cognitive function (e.g., less susceptible, or “healthier”) were more likely to enter the study at baseline, we would expect an underestimation of an adverse effect and possibly an apparent protective effect. It is also possible post-baseline attrition resulted in conservatively estimated effects of the highest (vs. lowest) aircraft noise category with cognitive decline, as those exposed to the highest aircraft DNL level and who had worse cognition were more likely to discontinue participation. Despite the exploratory nature, on the whole, these findings may indicate the need for noise abatement and mitigation efforts guided by the precautionary principle, which calls for action despite some uncertainty.^43–45^

Inquiry into the cognitive effects of noise in older adults is relatively new, with fewer studies investigating these in detail compared with other health endpoints; overall, results have been mixed.^2–6,10^ A common limitation is insufficient power (number of cases at high exposure levels). For example, a study in Sweden did not find associations between road noise and dementia, as only 6% of participants were exposed to high levels of road noise (≥55 dBA), and fewer were exposed to dementia cases (n = 17).^10^ Additionally, a study in the United States (Sacramento, CA) observed associations indicating a potentially elevated risk of cognitive impairment with higher road traffic noise levels, but this study was limited in sample size.^3^ Only two studies have examined both aircraft and road noise with cognitive impairment.^6,9^ Similar to our study, Wu et al^6^ assessed cognitive performance and the rate of change in performance. They found patterns similar to ours in their full models: with higher aircraft noise, global cognitive performance at baseline was slightly higher, yet the rate of decline was faster; there was also little evidence of an association between road noise exposure and cognitive decline.^6^ This is a common challenge in dementia research, as predictors of cognitive decline have been more difficult to observe than cognitive level, as later-life baseline cognition encompasses processes of cognitive decline at earlier ages.^46,47^ Our exposure time period may be later in life than what is etiologically relevant for dementia and cognitive decline.

Our work adds to the literature on the health effects of noise yet illustrates the critical need for extensive advances in noise-exposure assessment in the United States. We were limited to road traffic and aircraft noise estimates every 5 years from 1995 to 2015 as well as time-invariant community noise estimates, both calculated as 5-year time-weighted estimates, which may not fully represent long-term exposure. Furthermore, our exposure assessment for the analyses of cognitive function and decline was based on residential location at the time of the baseline visit; thus, it assumed that participants did not move during this exposure period. If a participant did move during the 5-year exposure period, this move would have introduced error into their measured exposure. However, measurement error emanating from changes in residential location was likely limited: when participants were asked about their residential stability in questionnaires administered during the 2000–2002 study cycle, most responded that they had been living at the same residence for 10 years or longer.

We also lacked data on local rail noise, which may be of concern for this area. Road noise estimates were obtained using data from the U.S. Federal Highway Administration’s Traffic Noise Model, which focuses on free-flowing noise and does not account for impulsive sounds, such as vehicle sirens/horns, loud mufflers, or sounds caused by poor car maintenance. Community noise estimates could capture these sounds, along with construction, music, and other outdoor noise; however, these noise samples were collected over short periods and did not include rush hour or nighttime periods.

Our source-specific road and aircraft estimates were modeled using noise propagation methods at a more highly resolved spatiotemporal resolution than what is currently publicly available, such as the U.S. DOT’s National Transportation Map. Additionally, we note that DOT advises against using the National Transportation Map to “evaluate noise levels in individual locations and/or at specific times,”^48^ likely because the methods used to track trends for major roads over time generate noise estimates that are too coarse for epidemiologic health effects research. Yet, despite the enhanced detail in the road noise models, our exposure data are likely still too coarse, as we did not integrate information on highly localized spatial features, such as the residential façade and building height. Not doing so contributes to measurement error, potentially masking adverse effects of road noise on dementia risk.^49–51^ For community noise in the United States, researchers have used the U.S. National Park Service model, which is derived from an extensive noise sampling campaign; however, these estimates are based on samples taken in more rural than urban areas, in which the model does not perform as well.^50,52,53^ Although our community noise estimates were derived from a land-use regression model incorporating kriging-based spatial interpolation methods, which do not account for the underlying physics of noise propagation, this model incorporated localized Chicago-specific data—including noise levels from a monitoring campaign—uniquely estimated exposure for each person and showed good predictive performance and temporal stability.

Lastly, additional dimensions of noise warrant a great deal of attention regarding dementia risk. For example, our exposure measures were annualized 24-hour averages, with penalties for nighttime noise (road and aircraft DNL), or were restricted to daytime nonrush-hour periods (community *L*_eq_). These averages smooth over temporal and other features of noise (e.g., pitch, timbre, texture). Nighttime noise may be particularly relevant to dementia etiology, given extensive evidence of the effects of noise on sleep^11,12^ and the influence of disordered sleep on dementia risk.^54,55^ Future research studying the effects of environmental noise should include samples with substantial variation in noise exposure, with a sufficient number of people and/or areas exposed to higher levels of noise.

This study presents a novel examination of the potential effects of environmental noise on cognitive function and dementia in a US community cohort of older adults, which is vital for informing US policy. Analyses were conducted within the context of a large, longitudinal cohort with almost 20 years of follow-up, a detailed collection of health-relevant personal characteristics, and exposure assessment of unique types of noise at the individual level. This study includes a greater proportion of Black individuals, who represent a historically understudied group with a greater risk of dementia.^56–59^ In addition, procedures used to diagnose AD and all-cause dementia were regular, uniform, and standardized. Moreover, the use of in-home assessments enhanced participation by enabling those with mobility or travel limitations to engage in this study.

## Conclusions

In this prospective study of older adults from a large US metropolitan area, we observed varying associations of environmental noise with cognitive functioning and dementia by noise source. Overall, this study emphasizes the need for improved characterization of noise in US cities, as well as highlights the nuances and complexities of source-specific noise, suggesting that transportation noise may have varying patterns and potential effects that could inform more focused and efficient mitigation strategies.

## ACKNOWLEDGEMENTS

We thank the CHAP participants and their families, investigators, and staff for their valuable contributions to this project.

## Supplementary Material


