# Supplementary material for: Residential aircraft and road noise exposure, cognition, and Alzheimer’s dementia in a US cohort of older adults
Source: Environ Epidemiol. 2026 Aug 10;10(4):e518. doi: 10.1097/EE9.0000000000000518 (PMC13461035; doi:10.1097/EE9.0000000000000518)
Supplement: Supplementary file 1 [file ee9-10-e518-s001.pdf]

Residential Aircraft and Road Noise Exposure, Cognition, and Alzheimer's Dementia in a United States Cohort of Older Adults

Stephanie T. Grady, Ryan M. Andrews, Junenette L. Peters, Sara D. Adar, Jonathan I. Levy, Todd Beck, Klodian Dhana, Pankaja Desai, Bernd J. Haupt, Joel D. Kaufman, Adam A. Szpiro, Robert S. Wilson, Kumar B. Rajan, Jennifer Weuve

eTable 1. Means and standard deviations of baseline cognitive scores, prior to standard normalization, in CHAP.

eTable 2. Multivariable-adjusted mean differences in cognitive performance for sub-scores (immediate recall, delayed recall, and MMSE) at baseline with increasing levels of aircraft and road noise.

eTable 3. Multivariable-adjusted mean differences in rate of change in cognitive performance for sub-scores (immediate recall, delayed recall, and MMSE) with increasing levels of aircraft and road noise.

eTable 4. Multivariable-adjusted mean differences in cognitive performance at baseline and 5-year rate of change with increasing levels of community noise.

eTable 5. Multivariable odds ratios and 95% CIs predicting attrition by aircraft noise exposure and other risk factors from pooled logistic regression model (n=9,950).

eTable 6. Multivariable odds ratios and 95% CIs predicting attrition by road noise exposure and other risk factors from pooled logistic regression model (n=9,950).

eFigure 1. Directed acyclic graph illustrating selective attrition in environmental noise and cognitive functioning analyses.

eText 1. Supplemental recruitment and statistical methods.

eText 2. Evaluation of potential attrition bias.

eReferences.

eTable 1. Means and standard deviations of baseline cognitive scores, prior to standard normalization, in CHAP.

Cognitive assessment	Scale	Mean (Standard Deviation)
East Boston Memory Test Composite recall (episodic memory)		
Immediate recall	0 – 12	8.43 (2.81)
Delayed recall	0 – 12	7.88 (3.14)
Modified Symbol Digits Modality Test (perceptual speed)	0 – 75	29.07 (14.38)
Mini-Mental State Examination (global function)	0 – 30	25.98 (5.25)
Composite global cognitive performance	average of the z-scores	0.034 (0.816)

Abbreviation: CHAP, Chicago Health and Aging Project.

eTable 2. Multivariable-adjusted[a] mean differences in cognitive performance for sub-scores (immediate recall, delayed recall, and MMSE) at baseline with increasing levels of aircraft and road noise.

Noise measure (per IQR)	Immediate recall		Delayed recall		MMSE	
	$N_{participants}$	Difference, SD units (95% CI)	$N_{participants}$	Difference, SD units (95% CI)	$N_{participants}$	Difference, SD units (95% CI)
Aircraft DNL category, dBA						
<45	4,421	Referent	4,398	Referent	4,452	Referent
45-49	2,958	-0.003 (-0.040, 0.033)	2,951	0.045 (0.002, 0.088)	2,983	-0.053 (-0.099, -0.007)
50-54	2,464	0.013 (-0.026, 0.052)	2,378	0.103 (0.056, 0.149)	2,482	0.025 (-0.024, 0.074)
≥55	729	0.009 (-0.052, 0.069)	718	0.084 (0.012, 0.157)	741	-0.028 (-0.104, 0.048)
Road DNL (per 8.5 dBA)	10,572	-0.033 (-0.063, -0.003)	10,526	-0.046 (-0.076, -0.015)	10,658	-0.038 (-0.070, -0.006)

[a] Adjusted for baseline year of visit, baseline age, race, sex, education level, averaged NO_x concentration, nSES score, time since baseline, and cross products of each covariate and time since baseline.

Abbreviations: CI, confidence interval; dBA, A-weighted decibels; DNL, day-night average sound level; IQR, interquartile range; MMSE, Mini-Mental State Examination; $N_{participants}$, number of participants; NO_x, nitrogen oxide; nSES, neighborhood socioeconomic status; SD, standard deviation.

eTable 3. Multivariable-adjusted[a] mean differences in rate of change in cognitive performance for sub-scores (immediate recall, delayed recall, and MMSE) with increasing levels of aircraft and road noise.

Noise measure (per IQR)	Immediate recall		Delayed recall		MMSE	
	N_{obs}/ $N_{participants}$	5-year rate of change, SD units (95% CI)	N_{obs}/ $N_{participants}$	5-year rate of change, SD units (95% CI)	N_{obs}/ $N_{participants}$	5-year rate of change, SD units (95% CI)
Aircraft DNL category, dBA						
<45	11,459 / 4,421	Referent	11,409 / 4,398	Referent	11,522 / 4,452	Referent
45-49	7,899 / 2,958	0.013 (-0.008, 0.034)	7,867 / 2,951	-0.003 (-0.030, 0.024)	7,964 / 2,983	0.031 (0.006, 0.056)
50-54	6,662 / 2,464	-0.009 (-0.031, 0.014)	6,644 / 2,378	-0.009 (-0.038, 0.019)	6,739 / 2,482	0.008 (-0.018, 0.034)
≥55	2,397 / 729	-0.048 (-0.089, -0.008)	2,378 / 718	-0.055 (-0.107, -0.002)	2,441 / 741	-0.030 (-0.078, 0.019)
Road DNL (per 8.5 dBA)	28,417 / 10,572	0.007 (-0.013, 0.026)	28,298 / 10,526	0.002 (-0.016, 0.021)	28,666 / 10,658	0.005 (-0.013, 0.022)

[a] Adjusted for baseline year of visit, baseline age, race, sex, education level, averaged NO_x concentration, nSES score, time since baseline, and cross products of each covariate and time since baseline

Abbreviations: CI, confidence interval; dBA, A-weighted decibels; DNL, day-night average sound level; IQR, interquartile range; MMSE, Mini-Mental State Examination; N_{obs}, number of observations; $N_{participants}$, number of participants; NO_x, nitrogen oxide; nSES, neighborhood socioeconomic status; SD, standard deviation.

eTable 4. Multivariable-adjusted[a] mean differences in cognitive performance at baseline and 5-year rate of change with increasing levels of community noise.[b]

Cognitive domain	N_{obs} / $N_{participants}$	Difference per IQR increment in community noise (2.6 dBA)	
		Baseline performance, SD units (95% CI)	5-year rate of change, SD units (95% CI)
Global cognition	27,003 / 10,359	-0.071 (-0.088, -0.054)	0.003 (-0.007, 0.012)
Perceptual speed	27,459 / 10,444	-0.051 (-0.066, -0.036)	0.005 (-0.004, 0.014)
Episodic memory	28,216 / 10,577	-0.061 (-0.079, -0.042)	0.006 (-0.005, 0.018)
Immediate recall	28,341 / 10,591	-0.058 (-0.076, -0.040)	0.009 (-0.003, 0.021)
Delayed recall	28,222 / 10,580	-0.059 (-0.077, -0.040)	0.006 (-0.006, 0.017)
MMSE	28,590 / 10,636	-0.064 (-0.084, -0.045)	-0.0112 (-0.0219, -0.0005)

[a] Adjusted for baseline year of visit, baseline age, race, sex, education level, averaged NO_x concentration, nSES score, time since baseline, and cross products of each covariate and time since baseline.

[b] Community noise estimated as L_{eq} per IQR (2.6 dBA).

Abbreviations: CI, confidence interval; dBA, A-weighted decibels; IQR, interquartile range; L_{eq}, equivalent continuous sound level; MMSE, Mini-Mental State Examination; N_{obs}, number of observations; $N_{participants}$, number of participants; NO_x, nitrogen oxide; nSES, neighborhood socioeconomic status; SD, standard deviation.

eTable 5. Multivariable-adjusted odds ratios and 95% CIs of attrition by aircraft and road noise exposure (n=9,950).[a]

**Covariate**	**Odds Ratios (95% CIs)**	
	**Model for Aircraft noise**	**Model for Road noise**
Age, yrs	1.08 (1.07, 1.08)	1.08 (1.07, 1.08)
Sex		
Female	1.00 *(Referent)*	1.00 *(Referent)*
Male	1.29 (1.22, 1.37)	1.30 (1.22, 1.37)
Race		
Not Black	1.00 *(Referent)*	1.00 *(Referent)*
Black	1.12 (1.02, 1.22)	1.09 (1.00, 1.19)
Cycle		
2nd	1.00 *(Referent)*	1.00 *(Referent)*
3rd	0.92 (0.86, 0.99)	0.92 (0.86, 0.99)
4th	0.65 (0.60, 0.72)	0.64 (0.58, 0.70)
5th	0.78 (0.69, 0.88)	0.77 (0.68, 0.86)
6th	0.55 (0.47, 0.64)	0.53 (0.45, 0.62)
Education, yrs		
<12	1.00 *(Referent)*	1.00 *(Referent)*
12	0.86 (0.80, 0.93)	0.86 (0.80, 0.93)
>12	0.72 (0.67, 0.78)	0.73 (0.67, 0.78)
nSES, average z-score	0.98 (0.97, 1.00)	0.98 (0.96, 0.99)
NO_x concentration, ppb	0.99 (0.99, 1.00)	0.99 (0.99, 1.00)
Aircraft DNL category, dBA		
<45	1.00 *(Referent)*	
45-49	1.05 (0.97, 1.13)	
50-54	0.99 (0.91, 1.07)	
≥55	0.97 (0.86, 1.09)	
Road DNL, dBA		1.01 (1.01, 1.02)

[a] Two models were implemented to examine predictors of attrition, with each model including either aircraft (model 1) or road (model 2) noise. Attrition was defined as either death or drop out of the study. Variables in both models taken from baseline (time-invariant) included sex, race, *APOE* ε4 carriership, and education; variables taken from visit prior to dropout, death, or end of study included age, cycle,

nSES, NO_x, global cognitive score, and (aircraft or road) noise. Continuous variables were modeled per 1 unit increase in the variable's respective measurement units.

Abbreviations: *APOE ε4*, apolipoprotein E ε4 allele; CI, confidence interval; dBA, A-weighted decibels; DNL, day-night average sound level; n, sample size; nSES, neighborhood socioeconomic status; NO_x, nitrogen oxide concentration; ppb, parts per billion; SD, standard deviation; yrs, years.

eTable 6. Joint associations of noise exposure and prior cognitive performance with attrition, multivariable beta estimates (log odds and log odds ratios) and 95% CIs of attrition: aircraft and road noise exposure (n=9,950).[a]

**Covariate**	**Log Odds Ratios (95% CI)**	
	**Model 1: Aircraft noise**	**Model 2: Road noise**
Intercept	-4.635 (-5.086, -4.184)	-5.434 (-6.340, -4.528)
Age, yrs	0.043 (0.037, 0.049)	0.043 (0.037, 0.049)
Sex		
Female	*Referent*	*Referent*
Male	0.226 (0.165, 0.287)	0.229 (0.168, 0.290)
Race		
Not Black	*Referent*	*Referent*
Black	-0.151 (-0.245, -0.057)	-0.167 (-0.261, -0.073)
Cycle		
2nd	*Referent*	*Referent*
3rd	-0.015 (-0.088, 0.058)	-0.018 (-0.091, 0.055)
4th	-0.295 (-0.389, -0.201)	-0.312 (-0.406, -0.218)
5th	-0.086 (-0.208, 0.036)	-0.098 (-0.220, 0.024)
6th	-0.394 (-0.555, -0.233)	-0.423 (-0.584, -0.262)
Education, yrs		
<12	*Referent*	*Referent*
12	0.046 (0.003, 0.089)	0.046 (0.003, 0.089)
>12	0.003 (-0.042, 0.048)	0.005 (-0.040, 0.050)
nSES, average z-score	-0.018 (-0.034, -0.002)	-0.021 (-0.037, -0.005)
NO_x concentration, ppb	-0.016 (-0.022, -0.010)	-0.019 (-0.025, -0.013)
Reverse-coded global cognitive score, SD units[b]	0.790 (0.731, 0.849)	0.967 (0.544, 1.390)
Aircraft DNL category, dBA		
<45	*Referent*	
45-49	0.109 (-0.034, 0.252)	
50-54	0.115 (-0.042, 0.272)	
≥55	-0.207 (-0.438, 0.024)	
Reverse-coded global cognitive score * Aircraft DNL category, dBA[c]		
<45	*Referent*	

45-49	-0.041 (-0.115, 0.033)	
50-54	-0.065 (-0.145, 0.015)	
≥55	0.116 (-0.009, 0.241)	
Road DNL, dBA		0.018 (0.004, 0.032)
Reverse-coded global cognitive score * Road DNL, dBA[d]		-0.003 (-0.011, 0.005)

[a] Two models were implemented to examine the multiplicative interaction term between global cognitive score and either aircraft (model 1) or road (model 2) noise exposure on attrition. Attrition was defined as either death or drop out of the study. Variables in both models taken from baseline (time-invariant) included sex, race, *APOE* ε4 carriership, and education; variables taken from visit prior to dropout, death, or end of study included age, cycle, nSES, NO_x, global cognitive score, and (aircraft or road) noise. Continuous variables were modeled per 1 unit increase in the variable's respective measurement units.

[b] We reverse coded global cognitive performance by subtracting each cognitive score value from 1 + the maximum score value; therefore, each standard deviation increase in this variable represented a 1-SD worse score for global cognitive performance).

[c] Multiplicative interaction term between each aircraft noise category and global cognitive performance score.

[d] Multiplicative interaction term between road noise and global cognitive performance score.

Abbreviations: *APOE ε4*, apolipoprotein E *ε*4 allele; CIs, confidence interval; dBA, A-weighted decibels; DNL, day-night average sound level; n, sample size; nSES, neighborhood socioeconomic status; NO_x, nitrogen oxide concentration; ppb, parts per billion; SD, standard deviation; SE, standard error.

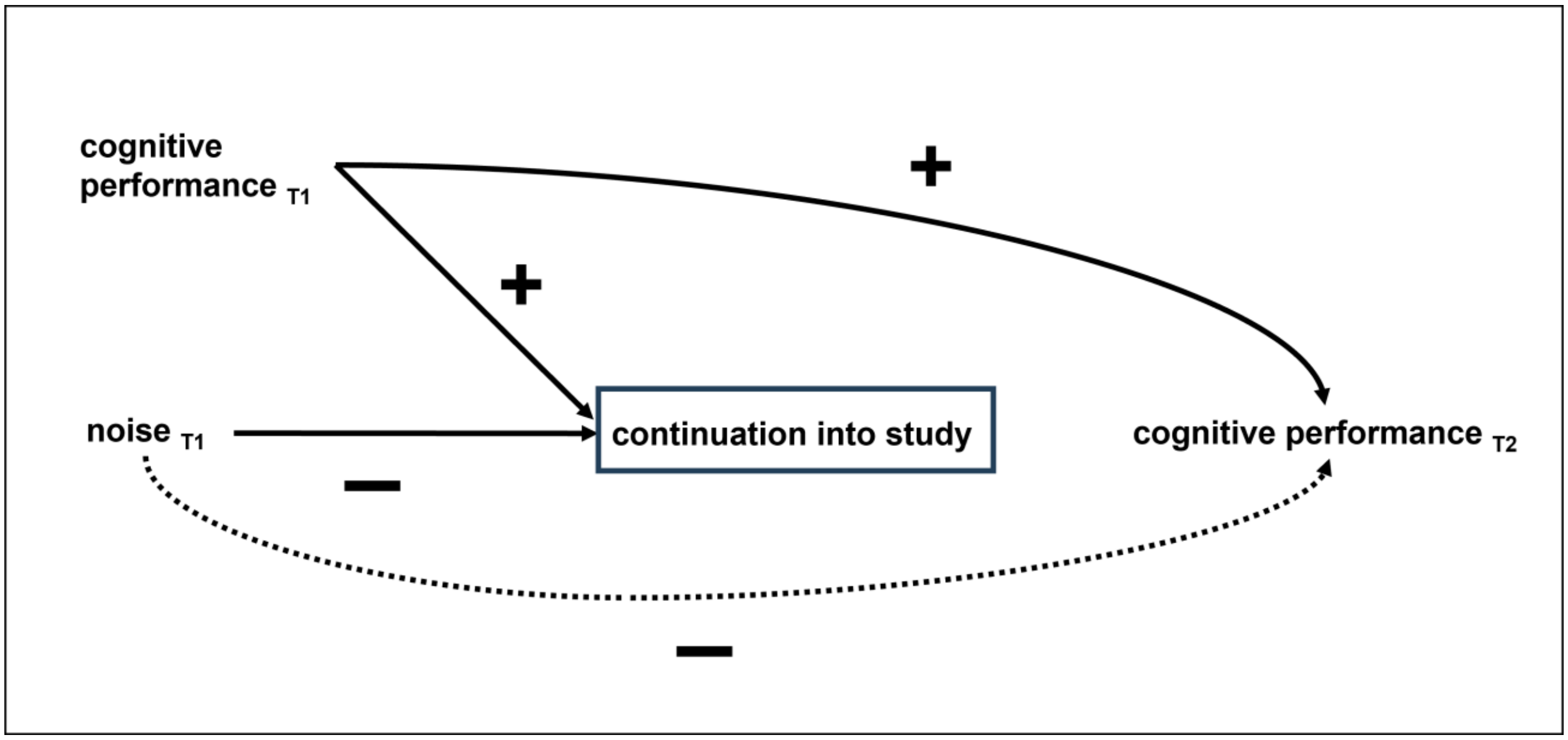

eFigure 1. Directed acyclic graph illustrating selective attrition in environmental noise and cognitive functioning analyses. In this figure, we depict the causal structure of how the association between environmental noise and cognitive decline (here, performance) may be influenced by selective follow-up through two time points of the study (time point 1 as T1 and time point 2 as T2) and the direction of the bias (positive, negative). We expect higher levels of environmental noise to result in decreased continued participation in the study (negative bias), while increased cognitive performance to increase continued participation (positive bias). Additionally, cognitive performance at T1 will directly influence cognitive performance at T2. Given these relations, we expect that the effect estimate of environmental noise on cognitive performance at T2 would have a negative bias, thus biasing effect estimates upwards (i.e., higher cognitive performance, slower cognitive decline). Abbreviations: T1, time point 1; T2, time point 2.

eText 1. Supplemental recruitment and statistical methods.

Study Recruitment

Participants were recruited from a door-to-door census of three adjacent neighborhoods (Washington Heights, Morgan Park, and Beverly) from 1993 to 1996, enumerating 7,826 individuals aged 65 or older.[1] Of these individuals, 6,158 (78.7%) agreed to enroll and participate in the baseline interview,[1] with successive cohorts recruited from residents who had reached age eligibility in 2000. In 2003, Mount Greenwood (neighborhood adjacent to Beverly and Morgan Park) became the fourth CHAP neighborhood.[1] Among the four neighborhoods, the closest international airport was located ~6 miles (9.7 kilometers) away. Participants who moved during the study period (19% of the sample) were included if their new residence was within Chicago city boundaries, the area covered by our exposure data.[2] Additionally, as our research question was related to long-term noise exposure, we created a 5-year time-weighted estimate of noise according to the road and aircraft noise data available for the participant's location during that 5-year period. We made the assumption that participants were residentially stable in the years prior to their enrollment. Participant moves after enrollment were recorded and incorporated in noise estimates computer after baseline (e.g., many of the 5-year estimates computed for the analyses of incident dementia).

Statistical Methods

To estimate the associations between noise and cognitive performance and decline, we fitted multivariable-adjusted linear mixed-effects models, using years since baseline as the time scale. The association of noise with baseline cognitive function corresponded to the coefficient for noise (β_1), and the association of noise with rate of cognitive change corresponded to the cross-product between noise and time (β_3), as written in the equation below:

$$Y_{ij} = \beta_0 + b_i + \beta_1 noise_{i1} + \beta_2 time_{ij} + \left(\beta_3 noise_{i1} time_{ij}\right) + \cdots + \beta_x covariates_{ij} + \left(\beta_{x+1} covariates_{i1} time_{ij}\right) + e_{ij}$$

where:

Y_{ij} = cognitive function for each participant *i* at time *j*

β_0 = intercept

b_i = random intercept for each participant *i*

β_1 = association of noise and baseline cognitive function

β_2 = association of time and baseline cognitive function

β_3 = association of noise and cognitive decline (rate of cognitive change)

β_j = associations of additional covariates and baseline cognitive function

β_k = associations of covariates and cognitive decline (rate of cognitive change)

e_{ij} = error term

eText 2. Evaluation of potential attrition bias.

Methods

Morbidity and mortality are common among older populations. This creates potential for bias in estimating the effect of cognitive change if both the exposure and risk of cognitive decline influence continuation in the study.[3–5] In studies of aging, continuation is commonly predicted by better cognitive performance.[3,6–8] In terms of our study, it is plausible that participants most susceptible to the adverse cognitive effects of noise may have experienced morbidity and mortality that prevented their continuation in the study. This differential attrition could bias estimated effects on cognitive change upwards, which would underestimate the adverse effects of noise on this outcome.

We evaluated this possibility by first estimating the association of exposure to each noise source with attrition regardless of attrition reason (dropout or death prior to end of study). For all models, if a participant missed a cycle and returned for a future cycle, we considered them "dropped out" at their first missed cycle. We used pooled logistic regression models (conditional on being alive and uncensored at the prior interview cycle), with one observation per participant per visit, adjusted for age (continuous), sex (female, male), race (Black, not Black), *APOE* ε4 allele carriership (carrier, not a carrier), education (<12 yrs, 12 yrs, >12 yrs), average NO_X concentration (continuous), nSES score (continuous), and cycle (2nd-6th interview cycle).

To evaluate the extent that noise exposure and prior cognitive performance (a predictor of cognitive decline) jointly influenced attrition, we fit additional models where we added terms for global cognitive score at the previous visit and the interaction between cognitive score and the noise measure.[9] In order to more easily interpret the direction of the joint association, we reverse coded global cognitive performance by subtracting each cognitive score value from 1 + the maximum score value (representing worse global cognitive performance with each standard deviation increase). A large non-zero coefficient for the interaction is consistent with high potential for selection bias; a positive coefficient indicates that attrition was more probable among those with both higher exposure and worse cognitive performance. If the coefficient was close to zero, we ended the evaluation for that noise measure. If the coefficient was

large, we would proceed to develop weights to adjust estimated effects of noise on cognitive change for selective attrition using the following methods. First, we fit a prediction model of attrition, again using pooled logistic regression models, but distinct in that these models were not built to estimate specific effects on attrition but rather to predict attrition as well as possible, using available variables from the cohort. In these models, we included predictors from baseline as previously described: sex, race, education level, and *APOE ε4* carriership. We also included potential predictors of attrition obtained at the visit prior to dropout, death, or end of study: noise level (aircraft or road), age, cycle, NO_x concentration, nSES score, self-rated health, self-reported diabetes, social network size, Nagi physical disability score, and global cognitive performance score. Self-rated health was operationalized as a rating scale for the question “As compared to other people your own age, would you say that your health is excellent, good, fair or poor?”. Social network size was calculated as the sum of the number of children, relatives, and friends that the participant had with monthly face-to-face contact.[10] This sum also accounted for the number of neighbors with whom the participant engaged in weekly friendly conversions.[10] The Nagi disability score represented the sum of components measuring physical activity performance of the upper and lower extremities; each component was rated on a 0 to 5 scale, where higher scores were indicative of less physical disability (greater ability).[11,12]

We used the logistic regression model c-statistic and Hosmer-Lemeshow goodness-of-fit test[13] to evaluate the goodness-of-fit and calibration of these prediction models. If a model predicted attrition well, we proceeded to develop inverse probability-of-continuation weights to use in analyses of cognitive change.

Results

We explored the joint associations of each source-specific noise measure and worse global cognitive performance prior to attrition or end of study (eTable 8). Here, we fit two models, one for each noise measure, and examined the direction and magnitude of the coefficients (represented as log odds and log odds ratios). In terms of aircraft noise, we observed small coefficients in diverging directions: coefficients

for the interaction (on the multiplicative modeling scale) between worse global cognitive performance and each of the middle noise exposure groups (45-49 dBA, 50-54 dBA) were negative, while the coefficient for the interaction between worse cognitive performance and the highest noise exposure group (≥55 dBA) was positive. The coefficients less than zero are consistent with higher attrition odds among participants with both higher exposure (i.e., not the lowest aircraft noise exposure category) and better cognitive performance, or with both low exposure and worse cognitive performance. Conversely, positive associations represented higher attrition odds among those with both higher exposure and worse cognitive performance. Shifting to road noise, the estimated coefficient for the multiplicative interaction term between road noise and cognitive performance was negative yet small, indicating that there may not be substantial selective attrition over follow-up.

Nevertheless, we attempted to generate a prediction model examining aircraft noise and study continuation (inverse of attrition), yet we were unable to obtain satisfactory goodness of fit. Although the Hosmer-Lemeshow test suggested good fit (χ^2=9.89, p-value=0.27), the c-statistic did not indicate strong predictive power (c-statistic = 0.71).

eReferences

1. Bienias JL, Beckett LA, Bennett DA, Wilson RS, Evans DA. Design of the Chicago Health and Aging Project (CHAP). *J Alzheimers Dis*. 2003;5(5):349-355. doi:10.3233/jad-2003-5501

2. Weuve J, D'Souza J, Beck T, et al. Long-term community noise exposure in relation to dementia, cognition, and cognitive decline in older adults. *Alzheimers Dement*. 2021;17(3):525-533. doi:10.1002/alz.12191

3. Weuve J, Tchetgen Tchetgen EJ, Glymour MM, et al. Accounting for bias due to selective attrition: The example of smoking and cognitive decline. *Epidemiology*. 2012;23(1):119-128. doi:10.1097/EDE.0b013e318230e861

4. Weuve J, Proust-Lima C, Power MC, et al. Guidelines for reporting methodological challenges and evaluating potential bias in dementia research. *Alzheimers Dement*. 2015;11(9):1098-1109. doi:10.1016/j.jalz.2015.06.1885

5. Banack HR, Kaufman JS, Wactawski-Wende J, Troen BR, Stovitz SD. Investigating and Remediating Selection Bias in Geriatrics Research: The Selection Bias Toolkit. *J Am Geriatr Soc*. 2019;67(9):1970-1976. doi:10.1111/jgs.16022

6. Hernán MA, Hernández-Díaz S, Robins JM. A Structural Approach to Selection Bias. *Epidemiology*. 2004;15(5):615. doi:10.1097/01.ede.0000135174.63482.43

7. Cole SR, Hernán MA, Margolick JB, Cohen MH, Robins JM. Marginal Structural Models for Estimating the Effect of Highly Active Antiretroviral Therapy Initiation on CD4 Cell Count. *Am J Epidemiol*. 2005;162(5):471-478. doi:10.1093/aje/kwi216

8. Andrews RM, Adar SD, Szpiro AA, et al. Association of Tailpipe-Related and Nontailpipe–Related Air Pollution Exposure with Cognitive Decline in the Chicago Health and Aging Project. *Environ Health Perspect*. 2024;132(12):127002. doi:10.1289/EHP14585

9. Mayeda ER, Tchetgen Tchetgen EJ, Power MC, et al. A Simulation Platform for Quantifying Survival Bias: An Application to Research on Determinants of Cognitive Decline. *Am J Epidemiol*. 2016;184(5):378-387. doi:10.1093/aje/kwv451

10. Ali T, Nilsson CJ, Weuve J, Rajan KB, de Leon CFM. Effects of social network diversity on mortality, cognition and physical function in the elderly: a longitudinal analysis of the Chicago Health and Aging Project (CHAP). *J Epidemiol Community Health 1979-*. 2018;72(11):990-996. Accessed November 14, 2025. https://www.jstor.org/stable/26896312

11. Nagi SZ. An epidemiology of disability among adults in the United States. *Milbank Mem Fund Q Health Soc*. 1976;54(4):439-467.

12. Weuve J, Kaufman JD, Szpiro AA, et al. Exposure to Traffic-Related Air Pollution in Relation to Progression in Physical Disability among Older Adults. *Environ Health Perspect*. 2016;124(7):1000-1008. doi:10.1289/ehp.1510089

13. Hosmer DW, Lemeshow S. Goodness of fit tests for the multiple logistic regression model. *Commun Stat - Theory Methods*. 1980;9(10):1043-1069. doi:10.1080/03610928008827941
